# Concurrent pigeon paramyxovirus-1 and *Acinetobacter baumannii* infection in a fatal case of pneumonia

**DOI:** 10.1080/22221751.2022.2054366

**Published:** 2022-03-30

**Authors:** Xiaohui Zou, Lijun Suo, Yiming Wang, Hongyun Cao, Shengrui Mu, Chao Wu, Lizhen Yan, Xiaowei Qi, Jianwei Lu, Binghuai Lu, Yanyan Fan, Hui Li, Lixue Huang, Lili Ren, Bo Liu, Bin Cao

**Affiliations:** a Department of Pulmonary and Critical Care Medicine, Center of Respiratory Medicine, National Clinical Research Center for Respiratory Diseases, China–Japan Friendship Hospital, Beijing, People’s Republic of China; b National Center for Respiratory Medicine, Beijing, People’s Republic of China; c Institute of Respiratory Medicine, Chinese Academy of Medical Sciences, Beijing, People’s Republic of China; d Department of Pulmonary and Critical Care Medicine, Zibo Municipal Hospital, Zibo, People’s Republic of China; e Zibo City Key Laboratory of Respiratory Infection and Clinical Microbiology & Zibo City Engineering Technology Research Center of Etiology Molecular Diagnosis, Zibo, People’s Republic of China; f Department of Clinical Microbiology, Zibo Municipal Hospital, Zibo, People’s Republic of China; g NHC Key Laboratory of Systems Biology of Pathogens and Christophe Mérieux Laboratory, Institute of Pathogen Biology, Chinese Academy of Medical Sciences & Peking Union Medical College, Beijing, People’s Republic of China; h Key Laboratory of Respiratory Disease Pathogenomics and Christophe Mérieux Laboratory, Chinese Academy of Medical Sciences & Peking Union Medical College, Beijing, People’s Republic of China; i Department of Respiratory Medicine, Capital Medical University, Beijing, People’s Republic of China; j Tsinghua University-Peking University Joint Center for Life Sciences, Beijing, People’s Republic of China

**Keywords:** Newcastle diseases virus, clinical metagenomics, pneumonia, cross-species transmission, pigeon paramyxovirus type 1

## Abstract

Pigeon paramyxovirus type 1 (PPMV-1), an antigenic variant of avian paramyxovirus type 1 (APMV-1), mainly infects pigeons. PPMV-1 genotype VI is the dominant genotype infecting pigeons in China. Human infection of avian paramyxovirus was rarely reported, and usually developed mild symptoms, such as conjunctivitis. We detected PPMV-1 in the lower respiratory sample from a fatal case with severe pneumonia; this patient aged 64 years presented cough, fever, and haemoptysis for 8 days and was admitted to hospital on Dec 26, 2020. He developed acute respiratory distress syndrome and sepsis in the following days and died of multiple organ failure on Jan 7, 2021. Sputum and blood culture reported multidrug-resistant *Acinetobacter baumannii* (ABA) for samples collected on days 22 and 19 post-illness, respectively. However, clinical metagenomic sequencing further reported PPMV-1 besides ABA in the bronchoalveolar lavage fluid. The PPMV-1 genome showed 99.21% identity with a Chinese strain and belonged to VI genotype by BLAST analysis. Multiple basic amino acids were observed at the cleavage site of F protein (113RKKRF117), which indicated high virulence of this PPMV-1 strain to poultry. The patient had close contact with pigeons before his illness, and PPMV-1 nucleic acid was detected from the pigeon feather. PPMV antibody was also detected in the patient serum 20 days after illness. In conclusion, concurrent PPMV-1 genotype VI.2.1.1.2.2 and ABA infection were identified in a fatal pneumonia case, and cross-species transmission of PPMV-1 may occur between infected pigeons and the human being.

## Introduction

Zoonotic diseases sporadically cross species barriers and cause human infection [1]. The avian-origin H5N1 and H7N9 avian influenza virus have caused several endemics in humans [2,3]. Newcastle disease (ND) is a highly contagious and diffusive disease that can cause high mortality in poultry [4]. Newcastle disease virus (NDV) is the causative pathogen of ND and belongs to avian paramyxovirus (APMV-1) in the genus *Avulavirus*, family *Paramyxoviridae* [5]. According to the latest update of the International Committee on Taxonomy of Viruses (ICTV), Paramyxovirus type 1(PMV-1) belongs to the genus *Avian Orthoavulavirus* 1 in the new sub-family, *Avulavirinae*, of the *Paramyxoviridae* family [6]. Pigeon paramyxovirus type 1 (PPMV-1) is an antigenic variant of APMV-1, which is mainly associated with infections of pigeons [7]. There is a considerable difference in APMV-1 strain virulence, ranging from lethal to asymptomatic infection in the farm poultry [8]. APMV-1 has been spread to Africa, Asia and parts of Central and South America and circulating as an enzootic disease[9]. China is one of the areas where APMV-1 infection frequently occurs in the poultry [10]. APMV-1 infects all major and minor species of domestic poultry, including chicken, ducks, pigeons, and so on. Pigeons are the natural hosts of APMV-1, playing an important role in the PMV-1 ecology [7]. In China, genotype VI is predominant in pigeons, and VI.2.1.1.2.2 contains the most recently isolated PPMV-1 viruses [11,12].

Generally, humans were not the susceptible hosts for APMV-1, though sporadic infection occurred in patients with occupational exposure to infected poultry [13]. Human infection of APMV-1 usually showed mild symptoms, and most of them resolved without clinical intervention [14]. However, severe lethal cases of APMV-1 were reported in immune-compromised patients after peripheral blood stem cells or allogeneic bone marrow transplantation [15–17]. Life-threatening APMV-1 infections in immune-competent persons have not been reported so far. We detected APMV-1 infection in a 64-year-old man with pneumonia after live pigeon exposure. His health deteriorated quickly, and the patient deceased after 21 days. An intensive clinical microbiology tests, including PCR/RT–PCR test of respiratory virus and microbiology culture of respiratory samples, showed no pathogens except *Acinetobacter baumannii* (ABA), which is multi-drug-resistant and is associated with severe adverse outcomes indicated by a previous study [18]. His bronchoalveolar lavage fluid collected on Jan 4 was sent to an In Vitro Diagnostics company for clinical metagenomic sequencing, which reported APMV-1 and ABA in the sample on Jan 9, 2021. We here detailed clinical demonstration and epidemiology investigation of this APMV-1 case in humans and proposed a possible pigeon–human transmission.

## Methods

### Clinical data and sample collection

The patient was admitted to the department of respiratory and critical care medicine, Zibo municipal hospital, Shandong, China, for the diagnosis of “respiratory failure, acute respiratory distress syndrome (ARDS), severe pneumonia” on Dec 26, 2020, 9 days post his illness onset (*poi*). Clinical information regarding clinical symptoms, clinical microbiology examination, and treatment was exported from electronic medical records. Intensive clinical microbiology tests, including influenza A/B RT–PCR test (nasopharyngeal swab on day 10 *poi*), sputum culture (day 9 and 20 *poi*), blood culture (day 19 *poi*), and an antibody panel, covering 11 respiratory pathogens (serum on day10 *poi*), PCR panel, covering 13 bacteria pathogens (sputum on day 10 *poi*), were conducted to detect the potential pathogens causing the disease. PPMV-1 antibody was measured using human NDV antibody ELISA test kit (Shjgogo, Shanghai, China) for the sample collected on day 20 *poi*, as extensive cross-reaction between PPMV-1 and NDV occurred in serum assays using polyclonal antisera [19].

### Epidemiology investigation

Field investigators and clinicians used a standardized surveillance reporting form to gather epidemiological data, including demographic characteristics, recent exposure to poultry, underlying medical conditions, and work environment on day 30 *poi* (9 days post-death). The patient was the doorman of the restaurant and helped the kitchen process live pigeons during his spare time. The pigeons were bought from a live poultry market and reared in the cages for 2∼4 days before processing. This breeding place was situated in the open backyard of the restaurant, tens of metres away from the kitchen. Environment samples in the restaurant, including cage swabs from where the pigeons were raised, and sewage in the backyard, were collected for the RT–PCR test of APMV-1 RNA; household samples, including water in the fish tank, cat feces from its hut, bedside excretion in the bedroom, were collected on the same day for APMV-1 detection. None of the restauran staff claimed close contact with the live pigeons or pigeon cages, and 3 of them provided serum samples for potential APMV-1 antibody testing; we also obtained the leftover serum from the Procalcitonin assay collected on day 20 *poi* for serology test.

### Clinical metagenomics and real-time RT–PCR

Bronchoalveolar lavage fluid (BALF) was mechanically lysed and homogenized using FastPrep 24 5G instrument (MP Biomedicals, USA). The lysate was separated into two aliquots and processed for DNA and RNA extraction, than library preparation through DNA/cDNA fragmentation, end-repair and A tailing, adaptor ligation and indexing, and PCR amplification. The qualified libraries were quantified and sequenced on the Ion torrent S5 plus platform (ThermoFisher, USA) with 200 bp of reads length.

Based on the APMV-1 matrix gene sequence assembled from clinical metagenomic sequencing data, we designed two primers, namely, NDV_M811_F: AGCGATGTACTCGGACCCTCTG, and NDV_M933_R: CAACCTGAGGGGAGGCATTTGCTA, to detect APMV-1 using the QuantiFast SYBR Green RT–PCR Kit on the QuantStudio 12 K Flex Real-Time PCR System (Thermofisher, USA).

### Bioinformatic analysis

Clinical metagenomic sequencing reads were analyzed using the CLC Genomics Workbench version 11.1. Briefly, the reads were first trimmed to filter them with Phred score <20 and length <100 bp. The qualified reads were then mapped to human reference GRCh38 to remove host-origin reads, followed by de novo assembly and BLAST alignment of the assembled contigs to the NCBI nt database. Based on the BLAST results, we download the hit with the highest score and the lowest E-value as reference for clean reads (non-host reads) mapping. A consensus sequence was extracted from the mapping result with each site covered by at least 5 reads. This consensus sequence was used to construct a phylogenetic tree using the MEGA 6 software [20], based on the phylogenetic classification system for APMV-1 [21].

To further investigate the pathogen spectrum of the sample, we aligned all the clean reads to the NCBI nt database using BLAST and visualized the taxonomy alignment using MEGAN 6 software, as described before [22].

## Results

### Patient and clinical symptoms

**Figure 1.**
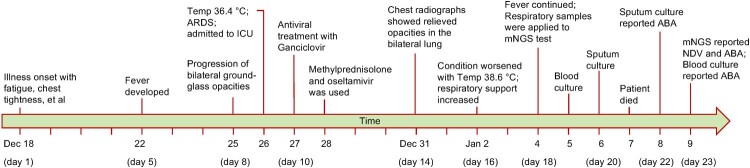
Timeline of the clinical course of the patient and identification of the causative pathogen.

**Figure 2.**
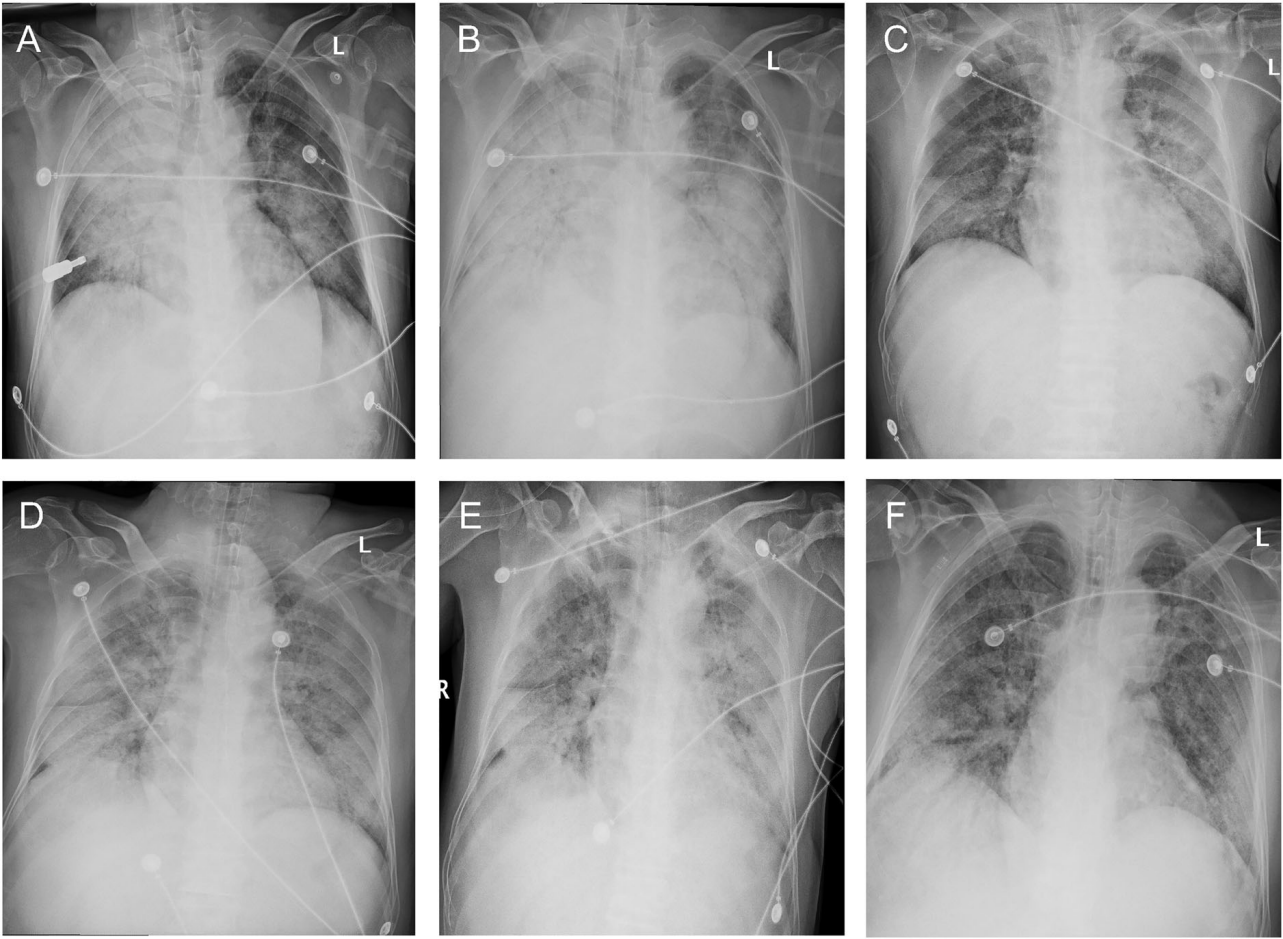
Chest radiographs during the clinical course. Bilateral ground-glass opacity and consolidation were observed on day 9 (Dec 26, Panel A) and worsened on day 11 (Dec 28, Panel B). Bilateral lung involvement was slightly relieved on day 14 (Dec 31, Panel C) after methylprednisolone was administrated on day 11. Bilateral ground-glass opacity and consolidation continued till day 16 (Jan 2, Panel D) and day 18 (Jan 4, Panel E) and relieved slightly on day 20 (Jan 6, Panel F).

The patient was a 64-year-old mans who denied any underlying conditions. He felt fatigued, chest tightness and vomited after dinner on Dec 18, 2020 (Figure 1) before being admitted to a local hospital. CT scan of the chest on Dec 20 (day 3 post-illness) showed right lung consolidation, with predominance in the peripheral upper lung zone (Figure S1A-C). The chest CT on day 8 showed worsening right lung consolidation and a new onset of ground-glass opacity in the left lung (Figure S1D-F). Because of severe pneumonia and he developed ARDS (Figure 2(A)); the patient was admitted to the intensive care unit on Dec 26, day 9 post-illness (Table 1).

**Table 1.** Clinical blood biochemistry tests and gas analysis.

	Normal range	Days after illness onset					
		3	9	11	14	17	20
Number of white blood cells (×10^9^ /L)	3.5-9.5	7.04	8.43	11.57		10.17	10.29
Proportion of neutrophils (%)	40–75		94.5	95.5	96	94	96.7
Proportion of lymphocytes (%)	20–50		3.7	3.2	2.5	3.8	1.7
Neutrophils count (×10^9^ /L)	1.8–6.3	6.26	7.97	11.05	9.06	9.56	9.95
Lymphocytes (×10^9^ /L)	1.1–3.2	0.44	0.31	0.37	0.24	0.39	0.18
C-reactive protein (nmol/L)	0–10			164.52	148.78	144.88	
Alanine aminotransferase(U/L)	9–50	37	30	29.8	45.8	39.6	42.6
Aspartate aminotransferase (U/L)	15–40	34	36	58.4	67.2	57	56.4
Alkaline phosphatase (U/L)	45–125			95	202.2	172.8	192
Total protein (g/L)	65–85		49	44.2	49.7	47.5	50.5
Globin (g/L)	20–40			21.5	22.9	22.4	23.4
Albumin (g/L)	40–55	32.5	24.7	22.7	26.8	25.1	27.1
D-dimer (mg/L)	0–0.55			4.7	> 20	14.8	
Uric Acid (μmol/L)	149–446			131	117		282
Creatinine (μmol/L)	44–105	80	62.5	59.44	62.5		123.7
Procalcitonin (ng/ml)	0–0.5		0.335	1.511	1.218	1.24	46.754
FiO_2_%	21–100		40	85	45	60	75
PaCO_2_ (mmHg)	35–45		35	59	51	43	108
PaO_2_ (mmHg)	80–100		67	55	63	57	58
PaO_2_/FiO_2_ (mmHg)	400–500		167.5	64.7	140	140	77.3
pH	7.35–7.45		7.46	7.29	7.3	7.47	7.16

Abbreviations: FiO_2_%: fraction of inspired oxygen; PaCO_2_: partial pressure of carbon dioxide; PaO_2_: arterial partial pressure of oxygen.

After admission, he received mechanical ventilation and anti-infection therapy with Imipenem/cilastatin sodium and moxifloxacin, plus ganciclovir started on day 10 (Table 2). His condition kept progressing, and more lung involvement was on the chest radiograph at day 11 (Figure 2(B)). On day 16, 2021, the patient’s disease worsened with the reappeared fever of 38.6°C and progression on the chest scan (Figure 2(D)). The patient has a meager count and proportion of lymphocytes during hospitalization. The fever continued till day 18, and no improvement was shown on the chest radiograph (Figure 2(E)); anti-infection therapy was then switched to vancomycin and micafungin (Table 2). After bedside bronchoscopy examination, 2 ml bronchoalveolar lavage fluid (BALF) was sent to routine bacteria culture and clinical metagenomic next-generation sequencing (mNGS). On day 20, the concentrations of aspartate aminotransferase and creatinine increased, indicating liver and kidney dysfunction. Despite chest radiograph showing relieved lung involvement (Figure 2(F)), metabolic acidosis occurred in the patient with a blood pH of 7.16 (Table 1). Despite comprehensive treatment of antibacterial, antiviral, and immunomodulatory, the patient had progressively worsened and presented refractory respiratory failure, multiple organ failure, and septic shock. Ultimately, he died 21 days post-illness onset (Figure 1).

**Table 2.** Complications, treatment, and clinical outcome of the patients.

Signs and treatment	
Fever	Yes
Temperature on admission (°C)	36.4
Highest temperature (°C)	39.8
Complications	
Failure of respiratory function	Yes
Septic shock	Yes
Heart failure	Yes
Liver failure	Yes
Oxygen treatment	Yes
Bacterial co-infection	Yes (*Acinetobacter baumannii* in blood culture)
Antibiotic treatment	Yes
Days 1–8	Moxifloxacin, ganciclovir, and then switched to cefperazone-Sulbactam and levofloxacin (does unknown)
Days 9–19	Imipenem (1 g given intravenously every 8 h)
	Moxifloxacin (one 0.4 g dose given intravenously)
Days 19–20	Vancomycin (0.5 g given intravenously every 12 h)
	Piperacillin/Tazobactam (4.5 g given intravenously every 8 h)
Day 21	Meropenem (1 g given intravenously every 12 h)
Glucocorticoid treatment	
Days 11–20	Methylprednisolone (40 mg given intravenously every 12 h)
Antiviral treatment	
Days 10–18	Ganciclovi (0.35 g given intravenously every 12 h)
Days 11–18	Oseltamivir (75 mg given orally twice a day)
Days 18–21	Arbidol (three 0.2 g doses given orally)
Antifungal treatment	
Days 19–20	Micafungin Sodium (one 100 mg dose given intravenously)
Intravenous albumin therapy	10 g (day 9-11)

### Clinical pathogen identification

**Figure 3.**
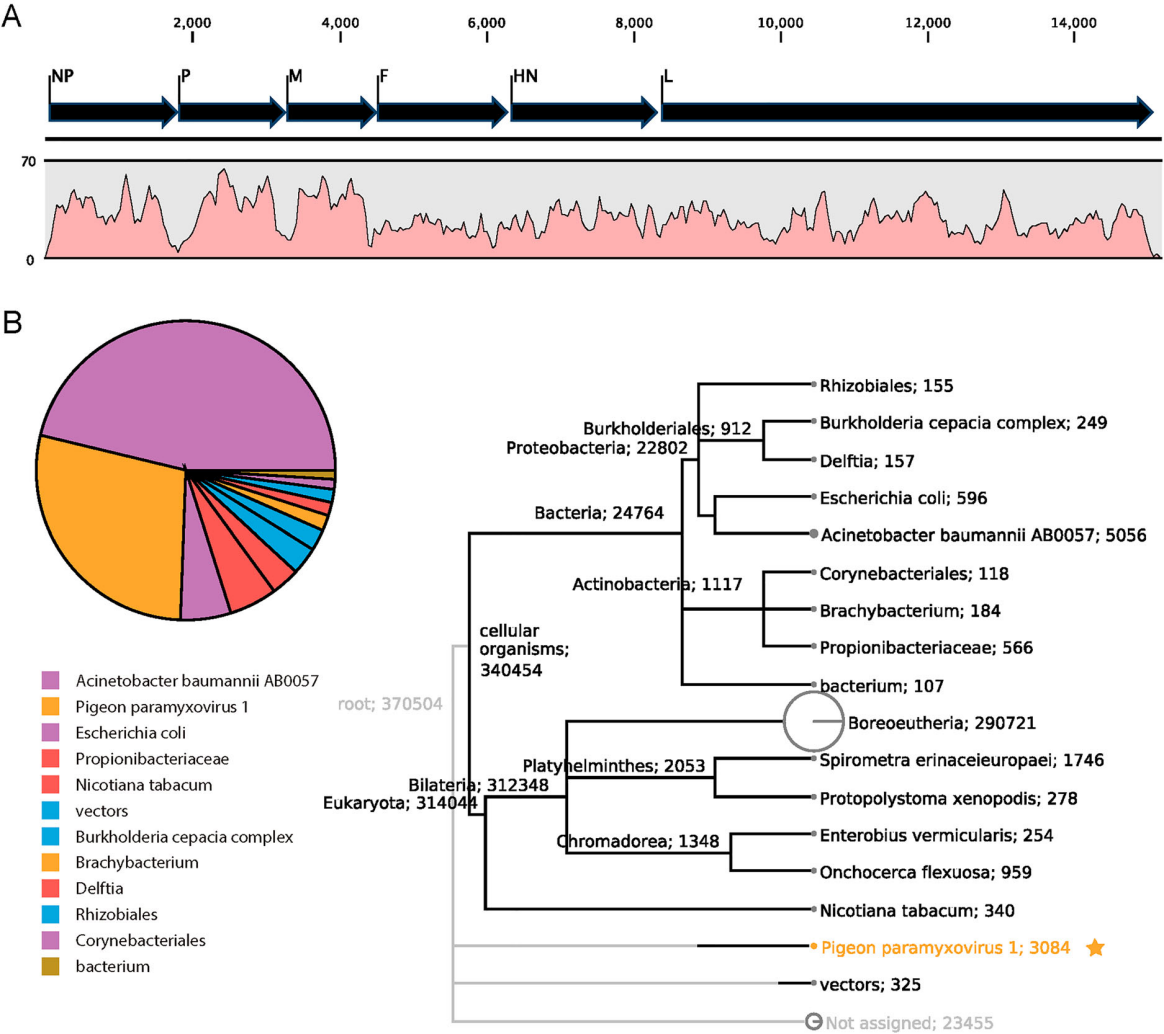
Metagenomic analysis of the clean reads after host sequences were removed. (A) The complete genome of reference APMV-1 strain pigeon/China/SDLC/2011 was fully covered by 3,289 reads with a mean depth of 31.2×. (B) taxa assignment of the clean reads using MEGAN software. Taxa and the number of their assigned reads were labelled in the polygenetic tree. The pie chart showed *Acinetobacter baumannii* contributed the most reads, followed by pigeon APMV-1 virus, among the reads of microorganism origin.

Sputum culture revealed no bacteria or fungi isolation on day 9. Serology tests conducted on day 10 detected no antibody against hepatitis B and C, EB virus, *Mycoplasma pneumoniae, Chlamydia pneumoniae*, respiratory syncytial virus, adenovirus, influenza virus A and B, parainfluenza virus A and B, coxsackievirus A and B, *Legionella pneumophila*, and echovirus. RT–PCR test of sputum on day 10 showed the patient was negative to influenza virus A/B (Dec 9 and 10) and a bacteria panel including 13 bacterial pathogens. However, the BALF collected on day 18 and blood collected on day 19 reported multidrug-resistant ABA by the culture on day 22. On day 23, clinical metagenomic sequencing reported 3,289 reads of avian avulavirus, which covered 97.73% of the complete genome of APMV-1 strain pigeon/China/SDLC/2011 (GenBank: JQ979176.1) in their pathogen database. Mapping results showed the mean depth was 31.2×, and all the APMV-1 genes were fully covered (Figure 3(A)). The laboratory performed mNGS had no history regarding any APMV-1 study or test; thus, there were low odds that the APMV-1 sequence was from environmental contamination.

The mNGS achieved 14,094,535 reads with a mean length of 145 bp, among which 9,780,257 (94.57%) were of human origin; after quality control processing, we obtained 416,672 clean reads for the downstream analysis. Besides APMV-1, mNGS also obtained 19,207 reads covering 39.3% of the genome of ABA strain UH9907. RT–PCR and sanger sequencing of APMV-1 fusion gene fragment confirmed APMV-1 in the nucleic acids extracted from the BALF sample. APMV-1 antibody was detected in the leftover serum collected on day 20 after routine blood biochemistry tests (Table 1). We further visualized the taxon origin of all the clean reads, using the metagenomic analysis software MEGAN. ABA contributed to most of the mNGS reads of microorganisms origin, followed by PPMV-1 (Figure 3(B)). Since we had not expected a rare viral infection of the patients and thus did not foresee the need to preserve clinical samples for further investigation, the patient’s clinical samples were disposed of as usual, and no samples were available for virus isolation or quantification.

### Virus genome analysis

**Figure 4.**
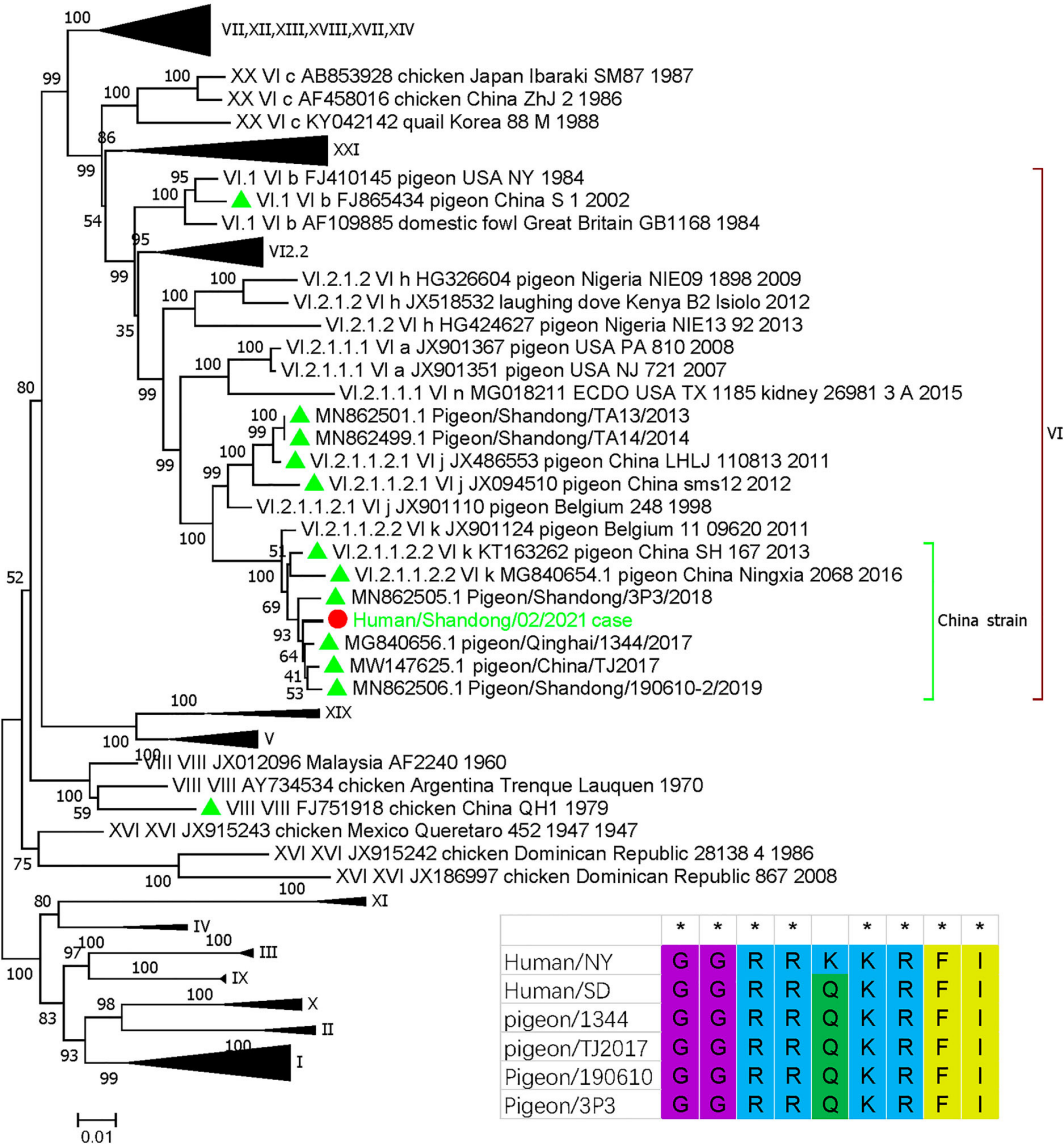
Phylogenetic classification of the APMV-1 virus using the full-length nucleotide sequence of the fusion gene, according to the phylogenetic classification system suggested by Dimitrov et al. [21]. The patient’s APMV-1 virus clustered with pigeon strains isolated in north China and belonged to genotype VI.2.1. Pigeon strains from China were labelled with a green triangle and the patient’s APMV-1 strain was labelled with a red circle. The cleavage sites of the fusion protein were aligned for several strains shown in the bottom-right corner, from which multiple basic amino acids were observed, indicating high virulence of the patient’s strain to poultry. Strain name abbreviations: Human/NY, Avian paramyxovirus 1 from the lethal human case of pneumonia reported by Goebel, et al. [15]; Human/SD, APMV-1 strain from our case occurred in Shangdong province in China; pigeon/1344, pigeon/Qinghai/1344/2017; pigeon/TJ2017, pigeon/China/TJ201; Pigeon/190610, Pigeon/Shandong/190610-2/2019; Pigeon/3P3, Pigeon/Shandong/3P3/2018.

The assembled APMV-1 genome we assembled was 15,013 nt long, with each site supported by at least 5 reads and covered all the ORF regions. BLAST analysis showed the virus had 99.21% identity to a pigeon strain isolated in eastern China (pigeon/Shanghai/1024/2019). In the phylogenetic tree, the patient’s virus clustered with a group of pigeon viruses isolated in north China, including a strain isolated from the same province in 2019 (Figure 4); the virus belonged to genotype VI.2.1.1.2.2, which mainly existed in the pigeons in China. Moreover, 113RKKRF117 motif was observed in the cleavage site of the fusion protein (Figure 4), which indicated the high virulence of this virus to poultry.

### Epidemiology investigation

A field investigation was conducted to explore possible cross-species transmission from pigeons to the patient. The patient was a doorman of a restaurant and helped the chef butcher poultry when the kitchen was short of hands. Five days before his disease onset, he butchered several pigeons and processed them in the backyard outside the kitchen, including plucking feathers and eviscerating internal organs before they were taken to the kitchen for cooking. The pigeons showed no clinical signs related to PPMV-1 infections, such as depression, ruffled feathers, or wing drop. On Jan 16, 2021 (Day 30 *poi*), the epidemiological investigators came to the restaurant to collect workplace environmental samples, including sewage in the backyard and pigeon feathers in the pigeon hut, and then came to his home to collect household samples, including cat faeces in the cat cage, water in the dish for cat, water in the fish tank, and bedside secretion contaminant. All the samples were negative in the RT–PCR test for APMV-1 except that one sample, the feather from the pigeon hut, was positive for APMV-1 RNA with a threshold value of 23.05. We further amplified a fragment of 535 bp in the F gene of this virus, and its sequence showed 100% identify to the patient’s APMV-1 F gene. However, the sera from the patient’s three companions, who claimed no contact with the pigeons, was negative for NDV antibody.

## Discussion

ND is a worldwide poultry infectious disease-causing endemics in poultry farms in many regions [4]. Sporadic ND outbreaks happen in China every year and mainly occur in chicken farms [23]. Human infection of APMV-1 was rare and usually caused mild illness or asymptomatic infections in immune-competent people [13]. The most frequently reported symptoms included eye disorders [24], usually consisting of unilateral or bilateral reddening, oedema of the eyelids, conjunctivitis and sub-conjunctival haemorrhage; acute keratoconjunctivitis was reported in one case coinfected with avian Newcastle virus and human adenovirus [25]; these symptoms are usually transient and most can recover without medical intervention. However, in immune-compromised person, APMV-1 infection can lead to a fatal outcome; two lethal cases of pneumonia were reported to be associated with APMV-1 infection in patients after peripheral blood stem cells/bone marrow transplantation [15,16]; another study reported fatal encephalitis caused by APMV-1 in a child after haematopoietic stem-cell transplantation[17]. We reported APMV-1 infection in a person without underlying conditions and developed fatal pneumonia.

Most human infections of APMV-1 were through direct contact with a virus or infected birds; mostly, laboratory workers, vaccinators, workers in the chicken plant are at high risk of getting an infection. Pedersden et al. reported significantly higher antibody titre against APMV-1 in people associated with poultry exposure [26]. In our case, the patient had close contact with the pigeon he processed; secondly, the pigeon carried APMV-1 with high titre in its feather collected on the hut, which showed 100% identify on the part of the F gene to the patient’s virus; whole-genome sequence of the patient’s APMV-1 showed high identify to pigeon strains and clustered with strains circulating on the local pigeons by phylogenetic analysis. It is very likely that cross-species transmission of APMV-1 happened between the pigeon and human. In Goebel’s report, the APMV-1 strain isolated from the patient with lethal pneumonia was also of pigeon origin [15]. The patient in our case reported no underlying conditions, and he was likely to be immune-competent when he was infected. The reason he got infection was possible because of his close exposure to the high titre of live APMV-1 virus when he butchered and processed the infected pigeon. Moreover, the pigeon APMV-1 strain in Goebel’s case induced pneumonia in experimentally infected cynomolgus macaques [27]. As the patient had a delayed admission to a professional hospital until day 9 *poi* and potent antimicrobial therapy before intensive clinical microbiology test, he may have missed the chance to detect causal agents or trigger microorganisms in the early stage of his diseases. Moreover, multi-drug-resistant *Acinetobacter baumannii* was cultured from the patient’s blood and sputum, which may contribute to the adverse outcome of the patient. The potential of APMV-1 to cause severe respiratory diseases in immune-competent person still need more evidence.

Generally, APMV-1 is not considered to be the cause of severe disease in humans and routinely, it is not the target on the prescribed diagnosis test. In Goebel’s case and ours [15], intensive microbiology tests were applied to detect tens of respiratory virus and bacteria pathogens but got no disease-causing agents. Because of the clear clinical signs of respiratory infection the patient showed, such as fever and lymphopenia which occurred in many severe viral infections [28,29], the patient’s BALF sample was applied to mNGS test and reported a large amount of APMV-1 reads, which covered the whole genome of the virus (Figure 3(A)). We further validated the mNGS result by RT–PCR and antibody test. Besides APMV-1, the mNGS also detected ABA in the sample, consistent with the culture result. mNGS here demonstrated its capacity to precisely detecte rare pathogens in severe cases when traditional methods failed. The ABA cultured in the BALF is multi-drug-resistant and appeared 11 days after ICU admission, which can be attributed to hospital-acquired infection and should not be the direct cause of the patient’s disease. Based on the clinical presentations, microbial detections, and significant increase in WBC counts, neutrophil proportion, and the level of CRP and PCT in the blood test, the patient’s disease progression seemed to be a combined effect of PPMV-1 and multi-drug-resistant ABA infection.

Lymphocytes are the main immune cells that eliminate the virus in viral infections. In conventional viral infections, the proportion of lymphocytes in the white blood cells usually increases at the acute phase of infection. Upon viral infections, the virus components are recognized by host-pathogen recognition receptors (PPRs) and trigger the innate immune signalling that finally induces the productions of various cytokines, such as interferons (IFN), which could act directly on CD8+ T cells and increase their abundance during the virus infection [30]. The increased lymphocytes, such as natural killer (NK) cells and cytotoxic T lymphocytes (CTLs), could produce cytokines and effector molecules to restrict viral replication and kill virus-infected cells [31]. However, in the late phase of severe viral infections such as COVID-19 and influenza [32,33], peripheral lymphocytopenia was observed, possibly due to redistribution and migration of lymphocytes to the respiratory system to resist the virus. The count and proportion of lymphocytes were quite low in the peripheral blood of our patient, which started on the third-day post-disease onset and persisted to his death. Depletion of lymphocytes was observed in many viral infections in humans and served as a risk factor for diseases deterioration, including avian influenza virus H7N9 [34], influenza virus pdmH1N1 [32], and SARS-CoV-2 [33]; lymphopenia may also indicate an adverse outcome of APMV-1 infection in humans.

mNGS offered the whole genome sequence of the APMV-1 virus related to patient’s disease. Polygenetic analysis showed this strain belonged to genotype VI.2.1.1.2.2, a genotype circulating in the pigeons in north China (Figure 4). Many studies have shown that multiple basic amino acids at the F0 cleavage site means high virulence of APMV-1 to chicken [35]; however, this is not always the case: some pigeon paramyxovirus type 1 strains cause minimal disease in chicken despite their F proteins having a multiple basic amino acid sequence [36,37]; the virulence of pigeon paramyxovirus type 1 does not always correlate with the cleavage site of its F protein [38]. We also found this motif in the strain that caused our case, which means this virus has a virulence potential in chickens. This motif was also presented in the APMV-1 strain detected in the feather sample through RT–PCR of F gene and Sanger sequencing. These data help establish the transmission route of pigeons to humans. Nevertheless, his 3 companions working in the same kitchen showed no symptoms or antibodies against the virus, indicating human-to-human transmission of this virus had not happened. The pigeons were temporarily reared in the backyard of the kitchen, which is an open place and has good ventilation; after feather plucking and evisceration here, the pigeon carcass was taken to the kitchen for cooking. Thus, the patient was the only staff who had close contact with the infected pigeon, which may explain why only one person was infected. However, we cannot exclude the possibility that host genetic traits played a role in the patient’s susceptibility to AMPV-1, such as the role of interferon-inducible transmembrane protein 3 (IFITM3) in the human influenza virus infection [39].

Our study had several limitations. First, the patient’s family did not permit an autopsy, so we have no autopsy specimens for histopathology or immunohistochemistry examinations to understand viral pathogenesis and the pathological lesions we were unable to expect the rare PPMV-1 infection in this patient, so no serum in the early infection phase was kept and thus cannot determine if there is a 4-fold increase in the antibody titres during the infections. Second, the pigeons were disposed before infected tissues could be sampled for virus isolation. Nevertheless, as we detected the complete genome sequences of APMV-1 in the patient’s BALF samples, adding APMV-1 sequence from feather, and the patient had 100% nucleotide identity based on F gene, the pigeon might be the source of infection.

In summary, this case of APMV-1 infection indicated that APMV-1 could be transmitted to humans through close contact with infected pigeons and caused fatal outcomes in people without underlying conditions. The APMV-1-caused severe pneumonia reported here is a rare event which challenges the clinical microbiology laboratory for accurate diagnosis. mNGS facilitated this uncommon pathogen identification in our case and should be considered in diagnosing severe infections of unknown origin. However, how APMV-1 caused severe human infection remains to be explored. This case stressed that personnel should wear personnel protective equipment when handling and processing potentially infected poultry.

## Supplementary Material

Supplemental Material

## Data Availability

Sequences are available via the National Genomics Data Center (BioProject number PRJCA006351under accession CRA004864).
