# Supplementary figures and images for: Concurrent pigeon paramyxovirus-1 and Acinetobacter baumannii infection in a fatal case of pneumonia
Source: Emerg Microbes Infect. 2022 Mar 30;11(1):968–77. doi: 10.1080/22221751.2022.2054366 (PMC8973364; doi:10.1080/22221751.2022.2054366)

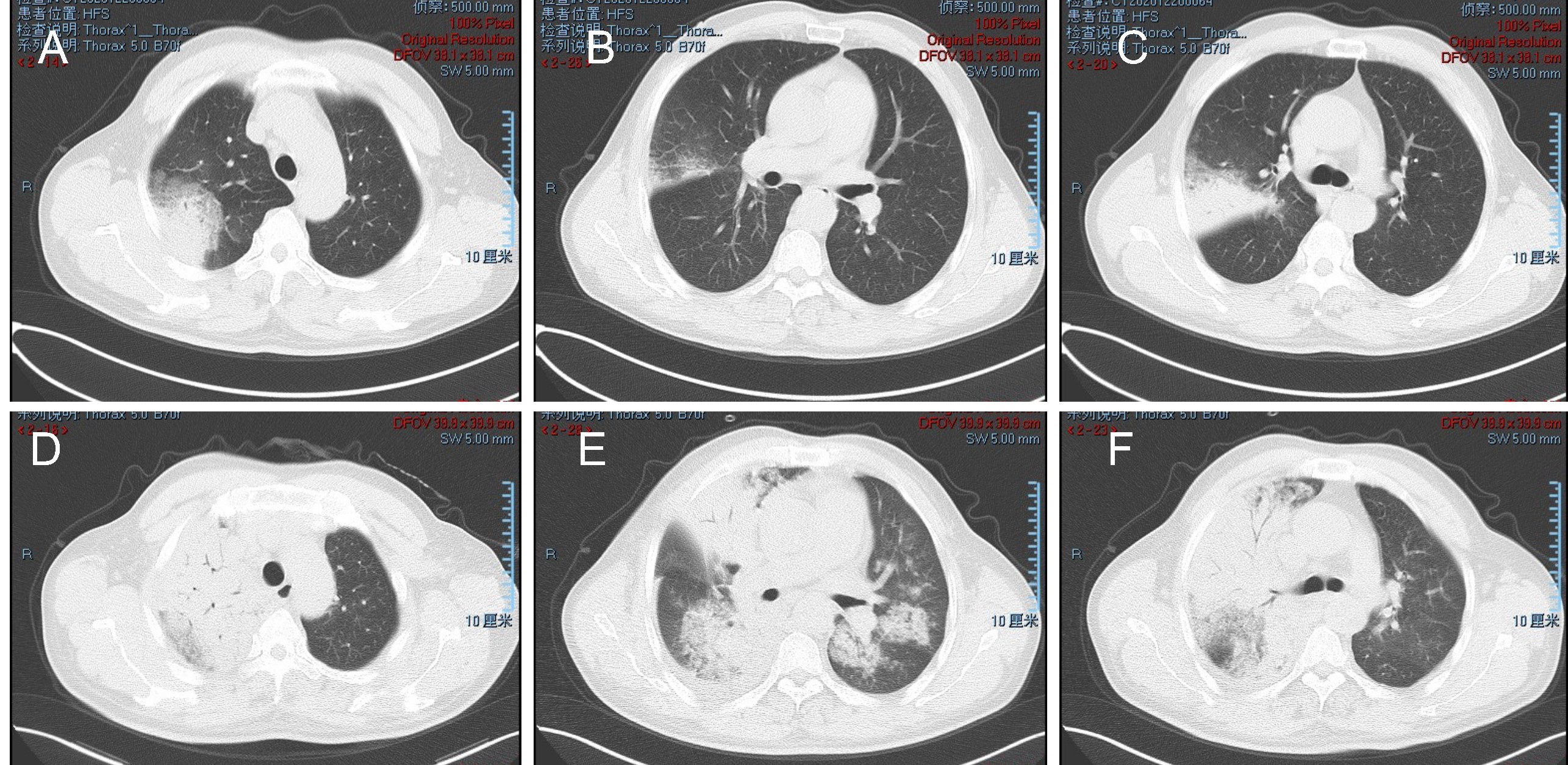

Supplement: Supplemental Material [file TEMI_A_2054366_SM2744.tif]
